# Enhanced Ferromagnetism and Tunable Magnetic Anisotropy in a van der Waals Ferromagnet

**DOI:** 10.1002/advs.202402819

**Published:** 2024-07-03

**Authors:** Xin Gao, Kun Zhai, Huixia Fu, Junxin Yan, Dongdong Yue, Feng Ke, Ying Zhao, Congpu Mu, Anmin Nie, Jianyong Xiang, Fusheng Wen, Bochong Wang, Tianyu Xue, Lin Wang, Hongtao Yuan, Zhongyuan Liu

**Affiliations:** ^1^ Center for High Pressure Science State Key Laboratory of Metastable Materials Science and Technology Yanshan University Qinhuangdao 066044 China; ^2^ Center of Quantum Materials and Devices and Chongqing Key Laboratory for Strongly Coupled Physics Chongqing University Chongqing 401331 China; ^3^ National Laboratory of Solid‐State Microstructures Jiangsu Key Laboratory of Artificial Functional Materials College of Engineering and Applied Sciences and Collaborative Innovation Center of Advanced Microstructures Nanjing University Nanjing 210000 China

**Keywords:** curie temperature, dome‐shaped phase diagram, high pressure, magnetic anisotropy, van der Waals ferromagnet

## Abstract

2D van der Waals (vdW) magnets have recently emerged as a promising material system for spintronic device innovations due to their intriguing phenomena in the reduced dimension and simple integration of magnetic heterostructures without the restriction of lattice matching. However, it is still challenging to realize Curie temperature far above room temperature and controllable magnetic anisotropy for spintronics application in 2D vdW magnetic materials. In this work, the pressure‐tuned dome‐like ferromagnetic‐paramagnetic phase diagram in an iron‐based 2D layered ferromagnet Fe_3_GaTe_2_ is reported. Continuously tunable magnetic anisotropy from out‐of‐plane to in‐plane direction is achieved via the application of pressure. Such behavior is attributed to the competition between intralayer and interlayer exchange interactions and enhanced DOS near the Fermi level. The study presents the prominent properties of pressure‐engineered 2D ferromagnetic materials, which can be used in the next‐generation spintronic devices.

## Graphical abstract

Spin controlling via various routes is one of the central themes in magnetic materials for spintronic device applications. With the application of high pressure, a dome‐like ferromagnet‐paramagnet phase diagram with the highest *T*_c_ fitted to be ≈480 K and continuously tunable magnetic anisotropy from out‐of‐plane to in‐plane direction is realized in an iron‐based 2D layered ferromagnet Fe_3_GaTe_2_.

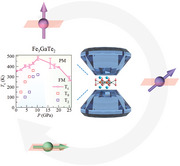

## 1 Introduction

The discovery of long‐range magnetic order down to the monolayer limit in vdW materials provides possibilities for constructing novel spintronic devices based on the Lego‐like stacked vdW heterostructures, expanding the device categories and accessing intriguing functionality.^[^ ^1^, ^2^, ^3^, ^4^, ^5^, ^6^ ^]^ Spin controlling via various routes (e.g., spin‐orbital coupling, exchange interactions, spin‐lattice coupling, and so on) has been one of the central themes in magnetic materials for spintronic device application.^[^ ^7^, ^8^, ^9^, ^10^ ^]^ The high tunability and relatively weak noncovalent vdW interlayer bonding make 2D layered magnetic materials susceptible to external stimuli, such as electrical gating, proximity effect, intercalation, pressure, and strain, offering effective approaches to tune the exchange interactions for Curie temperature enhancement and magnetic anisotropy modulation in these layered magnetic materials.^[^ ^11^, ^12^, ^13^, ^14^, ^15^, ^16^ ^]^

Among various vdW layered magnets, Fe_3_GaTe_2_ and Fe_3_GeTe_2_ have recently attracted considerable experimental efforts due to their high Curie temperature. They crystalize into a similar crystal structure with the space group of *P*6_3_/mmc, where the Ge (4s^2^4p^2^) was replaced by Ga (4s^2^4p^1^) in Fe_3_GaTe_2_, one less electron contributing to the itinerant ferromagnetism.^[^ ^17^, ^18^, ^19^, ^20^ ^]^ Surprisingly, the Curie temperature dramatically rises from 230 K for Fe_3_GeTe_2_ to 350–380 K for Fe_3_GaTe_2_. However, the hole substitution induced slight band structure shifting seems inadequate to explain the pronounced enhancement of Curie temperature in the counterpart Fe_3_GaTe_2_. Beyond the Stoner mechanism of ferromagnetism, recent experimental and theoretical works have revealed that both the itinerant and local magnetic moments account for the magnetism in the representative vdW ferromagnet Fe_3_Ge(Ga)Te_2_,^[^ ^21^, ^22^, ^23^, ^24^, ^25^ ^]^ where the local magnetic moment at each site is screened by the itinerant electrons. Within this context, high pressure, as an important thermodynamic parameter, can dramatically tune the electronic states, and serve as a tuning knob to reveal and control the interplay between localized and itinerant d‐electrons, which is not applied to Fe_3_GaTe_2_ to date.

In this work, we applied in situ high‐pressure electrical transport method to study the magnetic properties of vdW magnet Fe_3_GaTe_2_. By revealing the magnetism via anomalous Hall effect (AHE), magnetoresistance (MR), and Raman spectra under high pressure, we find that pressure is able to continuously tune the magnetic anisotropy from out‐of‐plane to in‐plane direction. Accompanying by the prominent magnetic anisotropy change, with the increase of pressure, the Curie temperature (*T*_c_) gradually enhanced to ≈480 K at *P* = 10.3 GPa, followed by gentle reduction with further compression, presenting a dome‐shaped ferromagnetic ‐paramagnetic phase diagram. Such behaviors are governed by the pressure‐engineered evolution of the electronic structure and magnetic exchange interaction. Our study presents the pressure tuning effect on magnetic properties for van der Waals magnet Fe_3_GaTe_2_ and the realization of ferromagnetism ≈480 K, which provide important clues for understanding ferromagnetism origin and potential application for 2D ferromagnet spin device.

## 2 Results and Discussion

**Figure 1:**
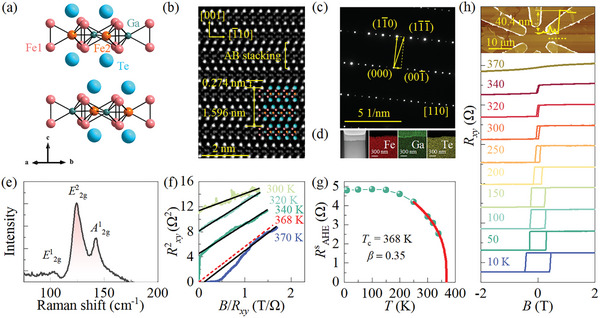
a) The schematic illustration of the crystal structure of Fe_3_GaTe_2_. b) The cross‐sectional HAADF‐STEM image and c) the corresponding SAED pattern of Fe_3_GaTe_2_. The atomic structure model is laid on the TEM image in (b). d) The dark field image and the corresponding elements mapping of Fe, Ga, and Te. The concentration of gallium in the protection layer is introduced during the focused ion beam (FIB) fabrication process. e) Raman spectra for Fe_3_GaTe_2_ at atmospheric pressure f) Arrot plot for the Hall resistance obtained from (h). The dashed line represents the Curie temperature obtained by interpolating the horizontal intercepts. g) Curve fitting by using the equation of $\alpha {\left(1-\left({\frac{T}{T}}_{c}\right)\right)}^{\beta }$ to obtain the critical exponent *β* and Curie temperature *T*_c_. h) Magnetic field dependence of transverse resistance *R_xy_* at different temperatures. The inset demonstrates the AFM image of the device with a sample thickness of 40.4 nm.

**Figure 2:**
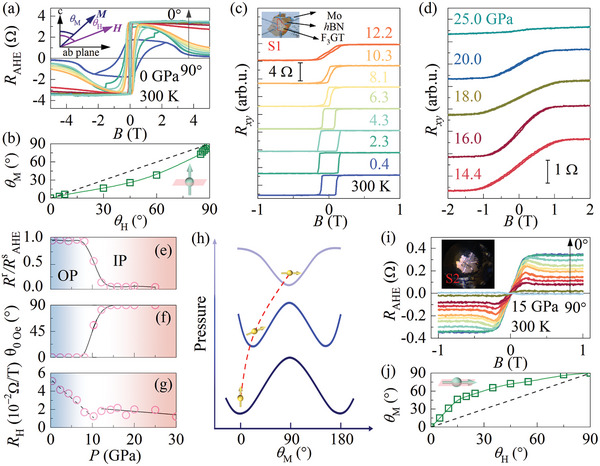
a) Anomalous hall resistance as a function of the magnetic field with varying angles. The inset shows the schematic illustration of the measurement geometry. b) *θ*_M_‐*θ*_H_ relationships obtained form (a), where $\theta_{M}=\;\arccos\left(\frac{R_{AHE}^{s}\left(\theta_{H}\right)}{R_{AHE}^{s}\left(\;\theta_{H}=0^{\circ }\;\right)}\right)$. Magnetic field dependence of hall resistance under pressures from (c) 0.4 to 12.2 GPa and (d) 14.4 to 25.0 GPa at 300 K. Pressure dependent of e) squareness ratio *R* ^r^ /*R* ^s^_AHE_, f) angle of magnetic easy axis *θ*_0 Oe_ and g) hall coefficient *R*_H_. h) Schematic illustration of the orientation of magnetic easy axis from out‐of‐plane to in‐plane depending on the local minima of free energy with increasing pressures. i) Angular dependence of *R*_AHE_ – *B* curve and j) corresponding *θ*_M_ – *θ*_H_ obtained at 15 GPa and 300 K.

**Figure** **1**a shows the schematic illustration of the crystal structure Fe_3_GaTe_2_. It processes a layered structure with centrosymmetric *P*6_3_/*mmc* symmetry for stoichiometric Fe_3_GaTe_2_. Two inequivalent Fe sites (Fe1 and Fe2) are located at the Fe1 layers and Fe2‐Ga layers within the Fe3Ga heterometallic slab, respectively, which is sandwiched by the upward and downward Te layers. Fe1 is surrounded by three Te ions, influenced by threefold crystal field potential. Figure 1b shows the transmission electron microscopy (TEM) image viewed from the [110] axis to give an insight into the atomic arrangement in the microscopic scale (Figure S1, Supporting Information for the view direction of [120]). The Fe_3_GaTe_2_ monolayer presents AB‐stacking periodicity along *c*‐axis with vdW gap *d* = 0.274 nm as illustrated in Figure 1b. Notably, Fe2 atoms deviate from their center position (mirror plane) along *c*‐axis. Concurrently, the forbidden (00*l*) (*l* = 2*n*+1, where *n* is integers) diffraction spots in *P6*_3_ */mmc* are observed as shown in the selected area electron diffraction (SAED) along [110] zone axis (**Figure** **2**c), as observed in recent report, indicating that the sample possesses nonstoichiometric ratio (Fe_3‐_ *_x_* GaTe_2_) with symmetry‐lowering structure of *P3m1*.^[^ ^26^ ^]^ Figure 1e shows the Raman spectra of Fe_3_GaTe_2_. Three Raman modes *E* ^1^_2g_ (103 cm^−1^), *E* ^2^_2g_ (124 cm^−1^), and *A* ^1^_1g_ (141 cm^−1^) can be observed, where *E* ^2^_2g_ mode represents the in‐plane vibrations of Fe, Ga, and Te and *A* ^1^_1g_ involves out of plane vibrations of Fe and Te.

We conducted Hall resistance measurements to study the magnetic transition under compression. Hall resistance (*R_xy_*) in a ferromagnetic material can be expressed by two components: ordinary Hall resistance (*R*_0_ = *R*_H_ *B*) and anomalous Hall resistance (*R*_AHE_ = *R*_s_ *M*), where *R*_H_, *R*_S_, and *M* represent the ordinary Hall coefficient, anomalous Hall coefficient, and magnetization, respectively. Figure 1h shows the temperature dependence of *R_xy_*‐*B* curves at ambient pressure for Fe_3_GaTe_2_ nanoflake. The obvious rectangular hysteresis loop can be observed below 340 K, indicating strong perpendicular magnetic anisotropy (PMA). We summarized the temperature dependence of the saturated *R*_AHE_ at *B*_sat_ = 4 T (*R* ^s^_AHE_) in Figure 1g. *R* ^s^_AHE_ is obtained by subtracting the linear component *R*_H_ *B* from *R_xy_*‐*B* curves and *R* ^s^_AHE_ is proportional to saturated magnetization. The extracted *R* ^s^_AHE_‐*T* curve is fitted with the critical power‐law form α(1 − *T*/*T_c_*)^β^, yielding *β* = 0.35 (satisfy to the critical exponents for the 3D XY model) and *T*_c_ = 368 K, comparable with previous results.^[^ ^27^ ^]^ Curie temperature is also determined by Arrot‐plot criterion for ferromagnetism as Figure 1f shown. By linearly interpolating the adjacent positive (340 K) and negative (370 K) intercepts obtained from the Arrot plots, the critical temperature (Curie temperature) was estimated to be *T* = 368 K when the intercept becomes zero.

For bulk Fe_3_GaTe_2_, the magnetic properties are examined by vibrating sample magnetometer (VSM) as presented in Figure S2 (Supporting Information). From the *M*‐*T* curve, the out‐of‐plane magnetization (*H* // *c*) is larger than the magnetic moment obtained from the in‐plane configuration (*H*⊥*c*). The magnetic hysteresis loops shown in Figure S2b,c (Supporting Information) confirm the PMA feature. Magnetic anisotropy energy density *K*_u_ can be estimated to be 5.64 × 10^5^ J m^−3^ at 300 K and 2.77 × 10^5^ J m^−3^ at 350 K by using the equation of $K_{u}\approx \frac{\mu_{0}M_{s}H_{an}}{2}$ (*M*_s_ is the saturated magnetization and *H*_an_ is the anisotropy magnetic field). In the case of the exfoliated nanoflake Fe_3_GaTe_2_, we perform angular‐dependent transverse Hall resistance measurements to investigate the magnetic anisotropy. By subtracting the linear part of magnetic field‐dependent ordinary Hall resistance (*R*_0_ = *R*_H_ *B*) from *R_xy_*‐*B* curves, we can obtain noncollinear *R*_AHE_‐*B* curves as shown in Figure 2a. The Stoner‐Wohlfarth model is widely used to describe the magnetic anisotropy of single‐domain magnets, which is applicable to the van der Waals magnet nanoflake.^[^ ^28^ ^]^ In this model, the free energy of a magnetic system involving magnetic anisotropy constants *K*_1_ and *K*_2_ can be written in the form of *E*  = *K*_1_ *sin* ^2^(θ_*M*_) + *K*_2_ *sin* ^4^(θ_*M*_) − *M_s_Bcos*(θ_*H*_ − θ_*M*_), where *θ*_H_ is angle between magnetic field and sample surface and *θ*_M_ denotes the angle between the magnetization and sample surface, respectively, as presented in the inset of Figure 2a. According to the Stoner–Wohlfarth model, for a given *H*, *M* was energetically stabilized at *θ*_M_ as shown in Figure 2h and the magnetization component along the *c* axis (*M*_c_) can be described as *M*_c_ = *M*_s_cos*θ*_M_. At *H* = 0, *M*_r_ corresponds to *M*_c_, and *θ*_0 Oe_ denotes the direction of spontaneous magnetization. In this way, we can use $\frac{M_{r}}{M_{s}}$ to approximately describe the direction of spontaneous magnetization axis (*M*_r_ denote remanent magnetization). Generally, the squareness ratio (sqr) of $\frac{M_{r}}{M_{s}}$ can define perpendicular magnetic anisotropy ($\frac{M_{r}}{M_{s}}\approx \;1$), in‐plane magnetic anisotropy ($\frac{M_{r}}{M_{s}}\approx \;0$) and canted magnetic easy axis ($\frac{M_{r}}{M_{s}}\approx \;0-1$), respectively.^[^ ^12^ ^]^ Precisely, *θ*_M_‐*θ*_H_ relationship can be derived from angular‐dependent AHE measurements at atmospheric pressure as shown in Figure 2b (refer to Supporting Information for details). By adopting the Stoner–Wohlfarth model to analyze *θ*_M_‐*θ*_H_ data at atmospheric pressure, we can obtain magnetic anisotropy *K*_1_ and *K*_2_ to be 4.87 × 10^5^ and −0.26 × 10^5^ J m^−3^, respectively, which correspond to the out‐of‐plane magnetic anisotropy (*K*_1_ > 0 and *K*_1_+*K*_2_ < 0).

Controlling magnetic anisotropy plays a key role in spin device applications. We further perform magnetotransport measurement by using a diamond anvil cell (DAC) to reveal the magnetic anisotropy evolution under high pressure. Figure 2c,d show the pressure‐dependent *R_xy_*‐*B* curves at *T* = 300 K. With the increasing of pressure from 0.4 to 8.1 GPa, the rectangular hysteresis loop representing strong PMA can be preserved. In contrast, with further increasing pressure from 10.3 to 25.0 GPa, the noncollinear *R_xy_*‐*B* curves without magnetic hysteresis appear, instead of rectangular hysteresis behavior. Notably, the coercive field increases from 1066 to 1390 Oe when increasing the pressure from 0.4 to 4.3 GPa, an indication of pressure‐enhanced ferromagnetism and PMA. The magnetocrystalline anisotropy affects the shape of hysteresis loops and determines the coercivity and remanence.^[^ ^28^ ^]^ We used the squareness ratio *R* ^r^/*R* ^s^_AHE_ (equivalent to the squareness ratio *M*_r_/*M*_s_, and *R* ^r^ is the Hall resistance at *B* = 0) to describe the rectangular shape as shown in Figure 2e. The *R* ^r^/*R* ^s^_AHE_ value remains unchanged at 1 up to 8.1 GPa. However, *R* ^r^/*R* ^s^_AHE_ decreases at *P* = 10.3 and 12.2 GPa, and eventually approaches 0 at higher pressure as demonstrated in Figure 2e. By applying the Stoner–Wohlfarth model with uniaxial magnetocrystalline anisotropy, the angles between the magnetic easy axis and *c* direction can be deduced as shown in Figure 2f, revealing that the magnetic easy axis rotates from out‐of‐plane to in‐plane under high pressure with canted magnetic easy axis at the intermediate pressure region. To understand this, Figure 2h illustrates the spin orientation state evolution with pressures, where the spin state is located at the minimum of free energy ($\frac{\partial F}{\partial \theta_{M}}=\;0$) mediated by magnetic anisotropy constant *K*_1_ and *K*_2_. To confirm high‐pressure magnetic anisotropy, the angular‐dependent *R*_AHE_‐*B* curves were performed at *P* = 15 and 21 GPa. Figure S3 (Supporting Information) shows the schematic illustration of the measurement setup. The corresponding *θ*_M_‐*θ*_H_ curves fall on the top‐left zone representing *θ*_M_ > *θ*_H_ with *K*_1_ < 0, indicating easy plane magnetic anisotropy for these two pressures (Figure 2; Figure S4, Supporting Information). The fitted magnetic anisotropy constants are −0.569 and −0.789 MJ m^−3^ for 15 and 21 GPa, respectively, when assuming a µ_0_ *MH* value of 1 MJ m^−3^ for the above two pressures, and the formula *F* = *K*_1_sin^2^θ_M_ − µ_0_ *HM*cos(*θ*_H_ − *θ*_M_) was used by considering first‐order constant of magnetic anisotropic energy to determine the magnetic easy axis.

To give an insight into out‐of‐plane to in‐plane magnetic transition and related electronic structure, pressure‐dependent Hall coefficient *R*_H_ at *T* = 300 K is summarized in Figure 2g. By fitting the high magnetic field linear component of *R_xy_*, Hall resistance *R*_H_ is obtained. With the increase of pressures, *R*_H_ monotonically decreases in the pressure regime of 0.4 GPa and 10.3 GPa. Above 10.3 GPa, the slope suddenly changes to a smaller value, consistent with the critical pressure of out‐of‐plane to in‐plane transition. Furthermore, negative to positive magnetoresistance occurs at the same critical pressure as presented in Figures S5 and S6 (Supporting Information). The above experimental results indicate a close relationship between Fermi surface reconstruction and magnetic anisotropy switch under high pressure.

**Figure 3:**
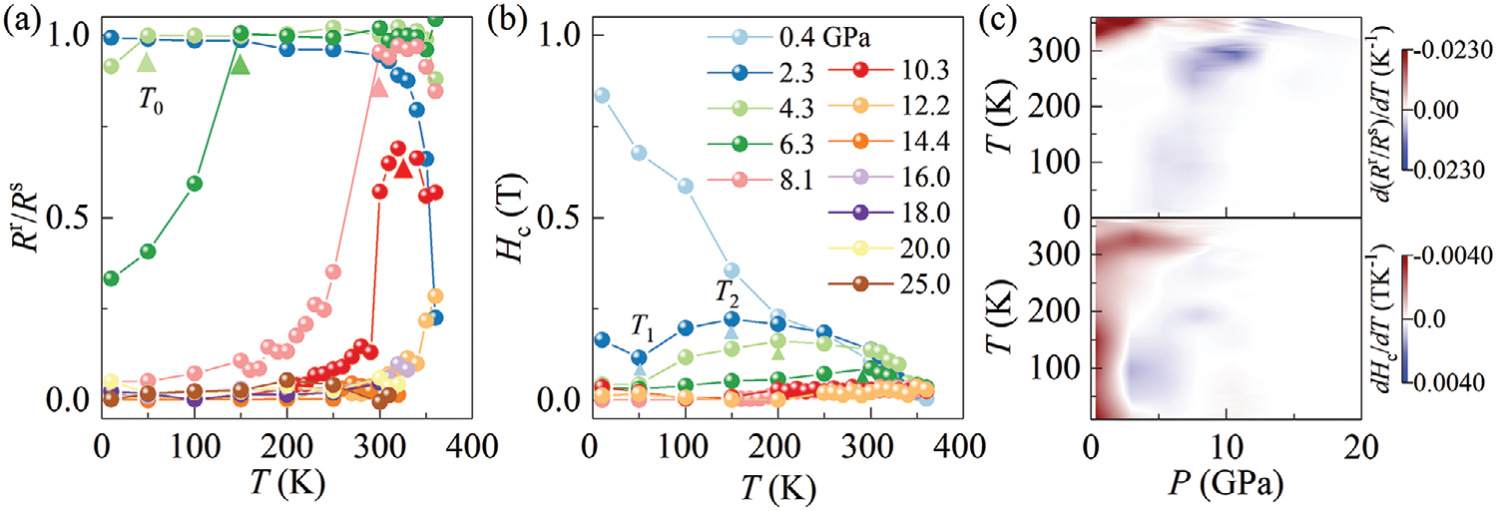
a) *R* ^r^/*R* ^s^ and b) coercive field *H*_c_ as a function of temperature at different pressures. c) The color plot of *d*(*R* ^r^/*R* ^s^)/*dT* and *dH*_c_/*dT* at the dimension of temperatures and pressures to distinguish the slope change.

To further clarify the evolution of the magnetic phase, transverse (*R_xy_*) and longitudinal (*R_xx_*) resistance of Fe_3_GaTe_2_ were measured at different pressures and temperatures. By extracting *R* ^r^/*R* ^s^_AHE_ from each symmetric *R_xy_*‐*B* curve presented in Figures S7 and S8 (Supporting Information), we can obtain *R* ^r^/*R* ^s^_AHE_ as a function of temperature at different pressures, as shown in **Figure** **3**a. For low pressures of 0.4 and 2.3 GPa, *R* ^r^/*R* ^s^_AHE_ was ≈1 below *T* = 300 K, indicating strong PMA, similar to atmospheric pressure characteristics. At 4.3 GPa, *R* ^r^/*R* ^s^_AHE_ derivate from 1 below 50 K defined as *T*_0_, and then reaches 1 with the increasing of temperature. Such behavior may be attributed to the magnetic easy axis turning from the canted angle to the out‐of‐plane direction. *T*_0_ shifts to a higher temperature with gradually increasing pressure to 8.1 GPa as guided by the arrows for the eyes. At *P* = 10.3 and 12.2 GPa, *R* ^r^/*R* ^s^_AHE_ values located between 0 and 1 at high temperatures originated from the small magnetic hysteresis features in *R_xy_*‐*B* curves. Above 12.2 GPa, no hysteresis feature in *R_xy_*‐*B* loops results in zero remanence with *R* ^r^/*R* ^s^_AHE_ corresponding to 0 in the whole temperature range, indicative of in‐plane magnetic anisotropy for Fe_3_GaTe_2_. To generally distinguish the magnetic phase regions separated by *T*_0_, the color mapping plot of *d*(*R* ^r^/*R* ^s^_AHE_) /*dT* at *T* and *P* dimensions is presented in the upper panel of Figure 3c. The *d*(*R* ^r^/*R* ^s^_AHE_)/*dT* > 0 and *d*(*R* ^r^/*R* ^s^_AHE_)/*dT* ≈ 0 region correspond to out‐of‐plane magnetic anisotropy and magnetic phase with canted spin orientation. The white area at the high‐pressure regime of the phase diagram with *d*(*R* ^r^/*R* ^s^_AHE_)/*dT* generally near to zero represents the existence of a new magnetic phase with in‐plane magnetic anisotropy.

To further confirm the evolution of magnetic anisotropy under high pressure, temperature‐dependent coercive field *H*_c_ at different pressures are summarized in lower panel of Figure 3c. At *P *= 0.4 GPa, *H*_c_ decreases with increasing of temperatures as shown in Figure 3b, which is a typical thermal‐dependent feature for magnetic materials. However, with increasing the pressure to 2.3 GPa, *H*_c_ first decreases with *T* warming to 50 K (*T*_1_), but reverses its trend and increases until up to *T* = 150 K (*T*_2_) occurring another drop. Below *T*_1_, a two‐step hysteresis feature of *R_xy_* (*B*) can be observed in Figure S8 (Supporting Information), which may be attributed to the high‐pressure induced intermediate magnetic phase at low temperature. Notably, above *T*_1_, the *H*_c_‐*T* displays a dome‐shaped curve presenting the maximum coercive field at *T*_2_. The shrinkage of *H*_c_ with the increasing of temperature for *T* > *T*_2_ indicates that the out‐of‐plane magnetic moment destabilized and spin orientation states stabilized at the canted angles. For *P* = 4.3 and 6.3 GPa, the decrease of *H*_c_ can also be observed at low‐temperature regions below *T*_1_. Besides, the critical temperature *T*_2_ shifts to higher temperatures with the pressure increases as shown in Figure 3b, where the color arrows are guides to the eyes for the *T*_2_ change. Further pressurized samples only show a small amount of coercive field above 300 K at 8.1 and 10.3 GPa as shown in Figure S8 (Supporting Information). To directly present the magnetic anisotropy‐related coercive field evolution under different pressures and temperatures, the phase diagram quantitively based on the derivative of *H*_c_(*T*) curve is summarized in Figure 3c. The magnetic phase with the out‐of‐plane magnetic anisotropy (*dH*_c_/*dT* > 0) and canted spin orientation (*dH*_c_/*dT* < 0) can be distinguished by the red and blue color region, respectively, as shown in Figure 3c, similar to upper panel of Figure 3c based on the derivation of *R* ^r^/*R* ^s^_AHE_‐*T* curve. The critical features like *T*_0_, *T*_1_, and *T*_2_ on *R* ^r^/*R* ^s^‐*T* and *H*_c_‐*T* curves under different pressures can also be reproduced on a different sample as shown in Figures S9–S11 (Supporting Information). Taken all together, the resultant anomaly features in the remnant magnetization and coercive field confirm the evolution of magnetic anisotropy under high pressure.

**Figure 4:**
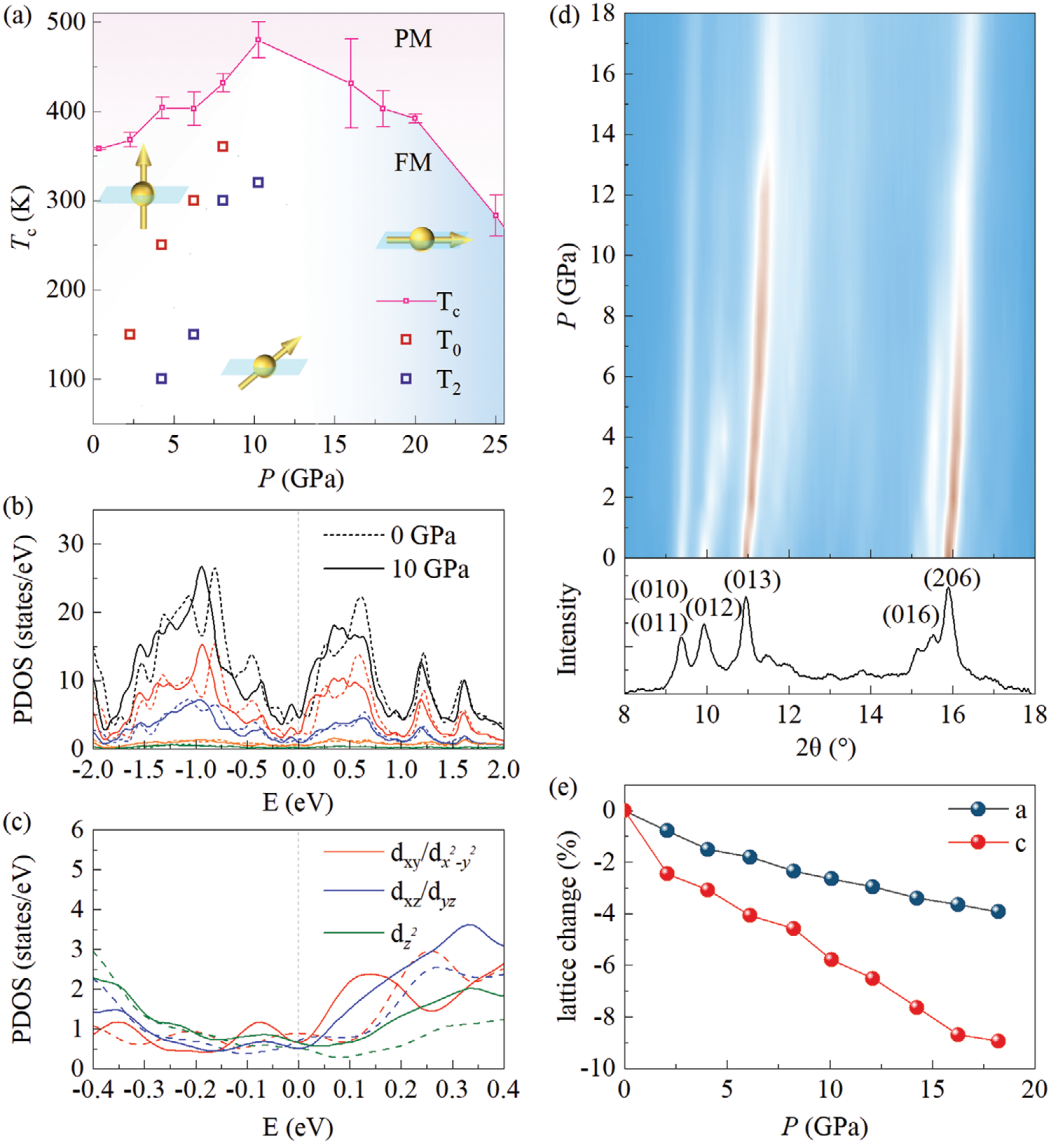
a) Temperature‐pressure magnetic phase diagram of Fe_3_GaTe_2_ by summarizing the magnetotransport data. *T*_c_s were determined by the Arrot‐plot method and the error bars are defined as the largest deviation from the average value of *T*_c_ with different fitting ranges. The arrows viewed from left to right denote the out‐of‐plane magnetic anisotropy, canted easy axis, and in‐plane magnetic anisotropy, respectively. b) PDOS for Fe_3_GaTe_2_ under 0 GPa (dashed line) and 10 GPa (solid line). The colors represent different contributions: black for total DOS, red for Fe1, blue for Fe2, olive for Ga, and orange for Te. c) PDOS around the Fermi level, projected onto the *d* orbitals of Fe atoms, is shown for both 0 GPa (dashed line) and 10 GPa (solid line). d) Color mapping of XRD pattern plotted as a function of diffraction angle and pressure for the powder sample Fe_3_GaTe_2_ at room temperature. The index of XRD diffraction peaks are presented in the lower panel. e) The relative change of crystal lattice ($a_{p}-a_{0}/a_{0}\;\mathrm{or}\;c_{p}-c_{0}/c_{0}$) under high pressure, where *a*_p_ (*c*_p_) is the lattice parameter at specific pressure and *a*_0_ (*c*_0_) represents the lattice parameter at atmospheric pressure.

A comprehensive phase diagram has been established by summarizing the magneto‐transport data as shown in **Figure** **4**a. Two approaches of Arrot‐plot and critical power law were applied to determine the Curie temperature as shown in Figures S12–S14 (Supporting Information). It should be noted that uncertainties can be introduced due to the limitation of the fitting temperature range (≈350 K) and further high‐temperature measurement is needed to obtain exact *T*_c_ values. However, the obtained Curie temperatures via the two independent methods are similar, thus guaranteeing that we capture the trend of *T*_c_ evolution under pressure. Detailed analysis of *T*_c_ estimation was presented in the supporting information. Two significant features should be addressed in the *T*‐*P* phase diagram. First, three magnetic phase regimes of out‐of‐plane anisotropy, canted spin orientation, and in‐plane anisotropy sequentially appear as the increase of pressures. Second, *T*_c_ can be gradually enhanced to ≈480 K at *P* = 10.3 GPa, followed by the decrease trend with further increasing of pressures, in sharp contrast to the continuous pressure suppression of *T*_c_ for the isostructural compound Fe_3_GeTe_2_. It is worth noting that the phase diagram based on the MR data (Figure S6c, Supporting Information) is also consistent with the temperature‐pressure (*T*‐*P* phase) magnetic phase diagram of Fe_3_GaTe_2_ shown in Figure 4a, revealing the hint of correlation between *T*_c_ and electronic band structure evolution.

From the viewpoint of itinerant FM, according to the Stoner criterion,^[^ ^28^ ^]^ pressure‐enhanced carrier density (as presented in Figure 2c) can help to promote magnetic ordering. Moreover, recent studies show that local moment and itinerant electrons both contribute to ferromagnetism, similar to the *f* electrons‐dominated heavy fermion system.^[^ ^22^, ^23^, ^24^, ^25^ ^]^ In such circumstances, the higher Curie temperature *T*_c_ (360 K) achieved in Fe_3_GaTe_2_ than that in isostructural Fe_3_GeTe_2_ (230 K) is possibly due to the stronger local moment effect in Fe_3_GaTe_2_. On the contrary, isostructural Ni_3_GeTe_2_ with suppressed effective electron mass (weak local‐moment ferromagnetism) present paramagnetism down to 2 K. These experimental facts show that localized electrons also play a crucial role in the formation of long‐range magnetic order in Fe_3_Ga(Ge)Te_2_. Considering interlayer and intralayer exchange interactions of localized magnetic moments (Fe1‐Fe1, Fe1‐Fe2, Fe2‐Fe2), these interactions may be described by Ruderman–Kittle–Kasuya–Yosida (RKKY) exchange. The RKKY exchange interactions are described by $J\;\left(r\right)=\;-J^{2}\times \frac{\sin\left(2k_{F}r\right)-2k_{F}\cos\left(2k_{F}r\right)}{{\left(k_{F}r\right)}^{2}}$, where the distance between two spins *r* and Fermi momentum of itinerant electrons *k*_F_ are associated with magnetic exchange interactions. Adopting the exchange interaction *J*(r) into the Hamiltonian, we can obtain $H\;=\sum_{i,j}J\left(r_{i},r_{j}\right)S_{i}\cdot S_{j}\;-\sum_{i}A{\left(S_{i}^{z}\right)}^{2}$(A represents the single‐ion anisotropy and *S* is the magnetic spin quantum number). The Curie temperature is given by $T_{c}=\;-\frac{2S(S+1)}{3}\left(\frac{1}{n}\sum_{i\neq j}J_{ij}+A\right)$ (*n* denote the number of nearest sites), where *J* parameters and A terms contribute to the ferromagnetic ordering temperature.^[^ ^21^, ^29^ ^]^ Under high pressure, the structural modulation affects the exchange interactions *J*(r), inducing the significant change of *T*_c_.

Raman mode A_1g_ involving the out‐of‐plane vibrations of Te and Fe show no apparent shift when compressing the sample (below 7 GPa) as demonstrated in Figure S15 (Supporting Information), suggesting the week van der Waals interlayer coupling. As revealed by the high‐pressure XRD (Figure 4d,e), the lattice shrinks with increasing of pressure and the *c*‐axis lattice is more compressible than the *a*‐axis lattice. Thus, the structure change leads us to speculate that *T*_c_ evolution stems from the competition of intralayer and interlayer exchange couplings. To gain a deeper understanding of *T*_c_ evolution under high pressure, DFT calculation was performed as shown in Figure 4b,c. The density of states (DOS) near the Fermi level (±0.15 eV) is observed to increase under pressure. The ferromagnetic ordering in Fe_3_GaTe_2_ is primarily attributed to the ferromagnetic exchange interactions *J*_1_ (Fe1‐Fe1) and *J*_2_ (Fe1‐Fe2), consistent with previous results.^[^ ^30^ ^]^ The nearest neighbor exchange interaction *J*_1_ predominantly arises from the Fe d_z_^2^ orbitals. The calculations reveal that, with the exception of the nearest neighbor Fe‐Fe distance corresponding to *J*_1_, most Fe‐Fe distances decrease under pressure. This reduction leads to an increase in the ferromagnetic exchange parameters, suggesting a higher Curie temperature (𝑇_𝑐_) under pressure. Additionally, the PDOS around the Fermi level projected on the Fe *d* orbitals generally increases under pressure (Figure 4c). Notably, the *d*_z_^2^ orbital shows a significant enhancement, attributed to the reduction in the *c*‐axis lattice constant. This increased *d*_z_^2^ PDOS reflects the enhanced interlayer overlap, which strengthens interlayer coupling and contributes to the observed changes in magnetic properties. Furthermore, the magnetic anisotropy energy (MAE) is also calculated by the force theorem method with spin‐orbit coupling (SOC), defined as MAE = *E*_in‐plane_− *E*_out‐of‐plane_. As shown in Figure S16 (Supporting Information), MAE decreases with the increase of pressure, indicating a gradual transition from out‐of‐plane to in‐plane ferromagnetism with increasing pressure.

## 3 Conclusion

In this work, high pressure‐tuned van der Waals ferromagnet Fe_3_GaTe_2_ undergoes a substantial change of magnetic anisotropy, from out‐of‐plane magnetic anisotropy to an intermediate canted spin state, and finally to the in‐plane magnetic anisotropy. Consequently, the *T*_c_ gradually enhanced to ≈480 K at *p* = 10.3 GPa, then decreased with the application of pressure, presenting a dome‐shaped ferromagnetic‐paramagnetic phase diagram. The pressure‐tuned intralayer and interlayer exchange interactions and enhanced DOS near the Fermi level are found to play a significant role in the dramatically enhanced *T*_c_.

## 4 Experimental Section

### Crystal Growth

Fe_3_GaTe_2_ was prepared by the self‐flux method. Fe powder (Aladdin, 99.99%), Ga (Aladdin, 99.9999%), and Te powder (99.999%) were weighted with the molar ratio of 1:1:2 and sealed in the vacuumed quartz tube. The mixture was heated to 1000 °C and the ingredients of the substance are expected to dissolve in a flux. After keeping the reactant at 1000 °C for 24 h, it cools to 880 °C in 1 h. Cooling the flux to 780 °C in 96 h, the quartz tube was subsequently quenched by ice water.

### AFM and TEM Characterization

The thickness of the mechanical exfoliated thin flake was measured by atomic force microscopy (Bruker Multimode 8). The Fe_3_GaTe_2_ nanosheet for TEM observation was manufactured with a focus ion beam (FIB, Helios G5, Thermo Fisher). TEM characterization was carried out using a JEM‐ARM300F2 microscope (JEOL, Ltd, Japan) at 300 kV.

### High‐Pressure Raman and XRD Measurement

The Fe_3_GaTe_2_ nanoflake (≈32 nm) was exfoliated from the bulk sample and transferred onto the diamond culet by using poly(dimethylsiloxane) (PDMS) stamps. The 4:1 mixture of methanol and ethanol was used as the pressure‐transmitting media. The in situ high‐pressure Raman spectra were conducted in a micro‐Raman microscope system (HR Evolution, Horiba) with an excitation wavelength of 532 nm. The pressure was determined by the ruby fluorescence line. The high‐pressure X−ray diffraction was performed at the Rigaku XtaLAB Synergy Custom diffractometer (Ag K_α_ radiation with λ = 0.5594 Å) by using a DAC loaded with Fe_3_GaTe_2_ powders. The detector (HyPix‐6000HE) with the pixel size of 100 µm × 100 µm was used to collect the diffracted X‐ray.

Device fabrication and high‐pressure transport measurement: Fe_3_GaTe_2_ nanoflake was mechanical exfoliated to 50–100 nm before being transferred to the diamond anvil culet (diameter: 400 µm) deposited with Mo electrode (thickness: 35 nm). An h‐BN flake capping layer was used to protect the Fe_3_GaTe_2_ from oxidation. The device fabrication process was conducted in a glove box filled with Ar gas (H_2_O, O_2_ < 0.1 ppm). Daphne 7373 was used as the pressure‐transmitting media. The electrical transport measurement was performed in a superconducting magnet (PPMS, EverCool II, Quantum Design Inc.) incorporated with our homemade measurement system. Hall resistance *R*_xy_ was obtained by using a double‐channel lock‐in amplifier (HF, Zurich Inc.), and four terminal resistance *R_xx_* was simultaneously acquired with a synchronized single‐channel lock‐in amplifier (MFLI, Zurich Inc.). The AC measurement frequency was set to 13.3 Hz. High‐pressure angular‐dependent electrical transport measurements were carried out in a cryogenic & magnetic field measurement system (ColdTube, MultiFields Technologies Co., Ltd.) providing a horizontal magnetic field up to 1.1 T and equipped with a sample probe rotating function.

First‐principles calculations were performed using the Vienna Ab initio Simulation Package (VASP) based on density functional theory (DFT) to understand the electronic properties in Fe_3_GaTe_2_ under pressure. The local Density Approximation (LDA) method was adopted for the exchange‐correlation function. The projector‐augmented‐wave (PAW) pseudopotential was implemented with an energy cutoff of 500 eV as the basis set. A hexagonal crystal structure with experimental lattice constants was used for both 0 and 10 GPa. In structural optimizations, all the atoms were fully relaxed until the residual force on each atom was less than 0.005 eV Å^−1^ within the energy convergence threshold of 10^–5^ eV. A Γ‐centered Monkhorst–Pack k‐point mesh of 15 × 15 × 3 was adopted for sampling the first Brillouin zone. The projected density of states (DOS) and magnetic anisotropy energy (MAE), defined as the energy difference between the in‐plane and out‐of‐plane magnetic configurations, were obtained with spin‐orbit coupling (SOC) considered.

## Conflict of Interest

The authors declare no conflict of interest.

## Supporting information

Supporting Information

## Data Availability

The data that support the findings of this study are available from the corresponding author upon reasonable request.
