# Supplementary material for: Enhanced Ferromagnetism and Tunable Magnetic Anisotropy in a van der Waals Ferromagnet
Source: Adv Sci (Weinh). 2024 Jul 3;11(33):2402819. doi: 10.1002/advs.202402819 (PMC11434145; doi:10.1002/advs.202402819)
Supplement: Supplementary file 1 — Supporting Information [file ADVS-11-2402819-s001.docx]

**Supporting Information**

Xin Gao^1^, Kun Zhai^1,*^, Huixia Fu^2,*^, Junxin Yan^1^, Dongdong Yue^1^, Feng Ke^1^, Ying Zhao^2^, Congpu Mu^1,*^, Anmin Nie^1^, Jianyong Xiang^1^, Fusheng Wen^1^, Bochong Wang^1^, Tianyu Xue^1^, Lin Wang^1^, Hongtao Yuan^3,*^, Zhongyuan Liu^1^

1. **TEM image viewed from [120] direction and the corresponding SAED**
2. **Magnetic properties for bulk Fe_3_GaTe_2_**
3. **Magnetic antitropy energy obtained from angular dependent hall resistance measurement based on Stoner-Wohlfarth model**
4. **Magnetic anisotropy under high pressure**

**5. Hall resistance and magnetoresistance at different temperatures and pressures**

**6. Reproducibility of *T*_c_ enhancement and magnetic anisotropy evolution under pressure**

**7*. T*_c_ determination**

**8. High pressure Raman spectroscopy**

**9. Magnetic anisotropy energy calculation**

**1. TEM image viewed from [120] direction and the corresponding SAED**

**
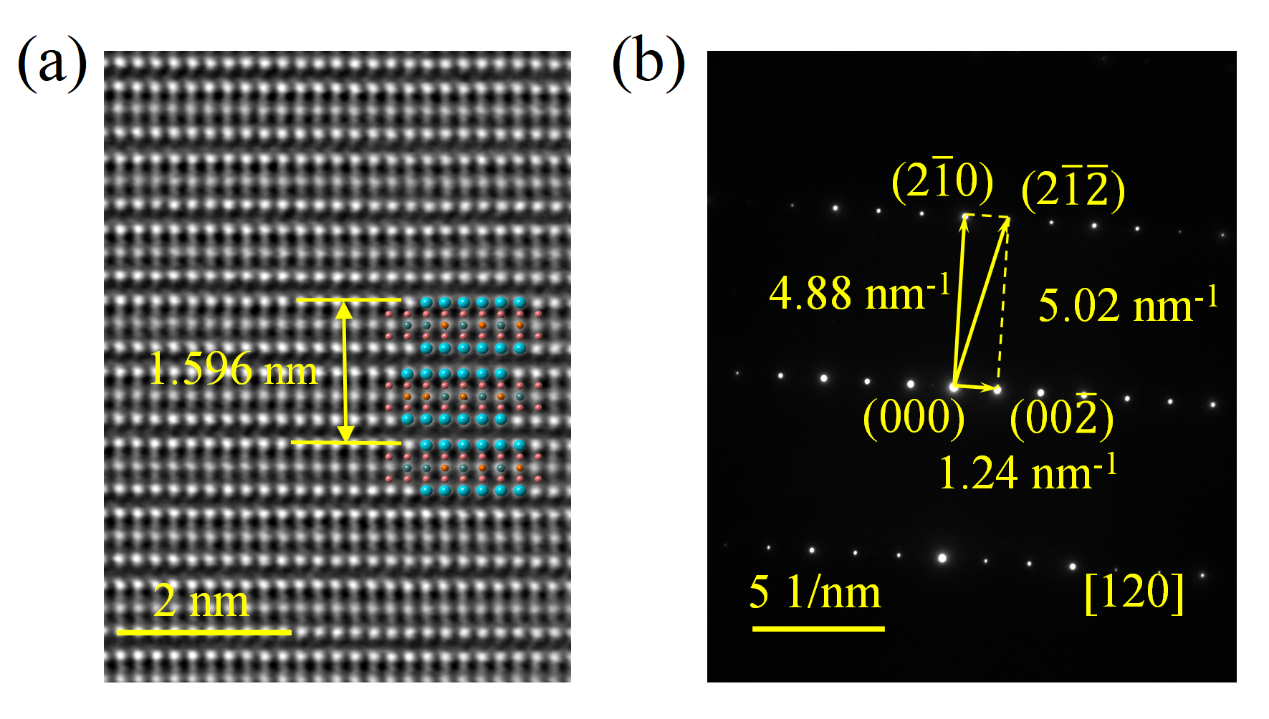
**Figure S1 (a) Magnified HAADF-STEM image viewed at [120] and (b) the corresponding SAED pattern of Fe_3_GaTe_2_.

**2. Magnetic properties for bulk Fe_3_GaTe_2_**


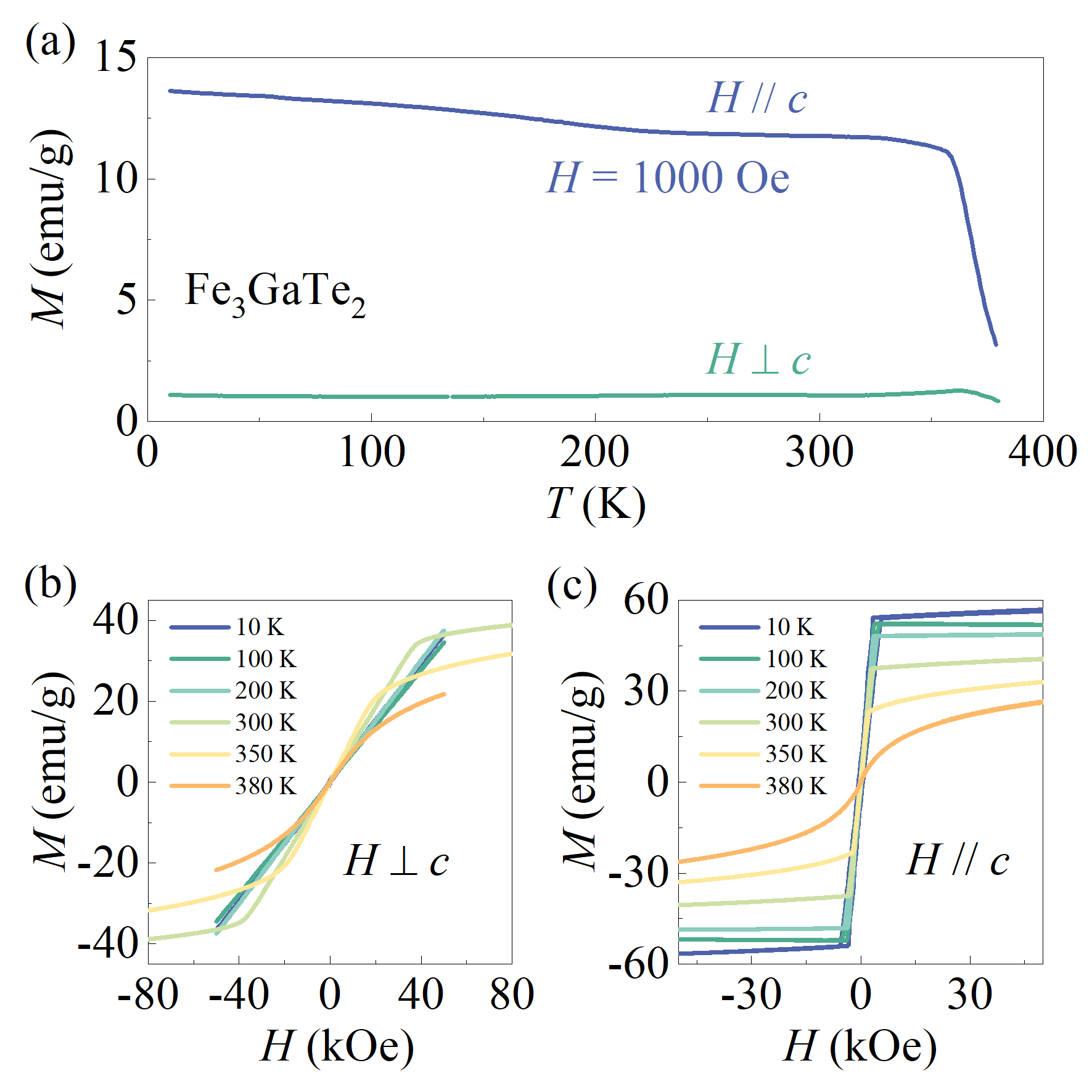


Figure S2 (a) Temperature dependence of magnetization along *c* axis and perpendicular to *c* axis after field cooling process with *H* = 1000 Oe. Isothermal hysteresis loops with (b) *H* perpendicular to *c* axis and (c) along *c* axis. The bulk magnetization measurements were performed in PPMS (Ever Cool II) equipped with a vibrating sample magnetometer (VSM). The anisotropy magnetic field can be determined to be *H*_an_ = 37800 Oe at *T* = 300 K and *H*_an_ = 23000 Oe at 350 K.

**3. Magnetic antitropy energy obtained from angular dependent hall resistance measurement based on Stoner-Wohlfarth model**

Fitting magnetic hysteresis loop with Stoner–Wohlfarth model.

The total energy of system can be described by the combination of magnetic anisotropy energy and Zeeman energy with the consideration of first order magnetic anisotropy energy *K*_1_ and second order magnetic anisotropy energy *K*_2_.

$\frac{E}{V}=K_{1}{sin}^{2}\left( \varphi-\theta\right)-M_{s}Bcos\varphi$ (S3-1)

φ is the angle between magnetic field and magnetic moment. θ is the angle between the magnetic field and the magnetic anisotropy.

By deriving of *E* by φ, we can obtain:

$\frac{\partial E}{\partial\varphi}=2K_{1}\sin\left( \varphi-\theta\right)\cos\left( \varphi-\theta\right)+M_{s}Bsin\varphi=0$ (S3-2)

**4. Magnetic anisotropy under high pressure**


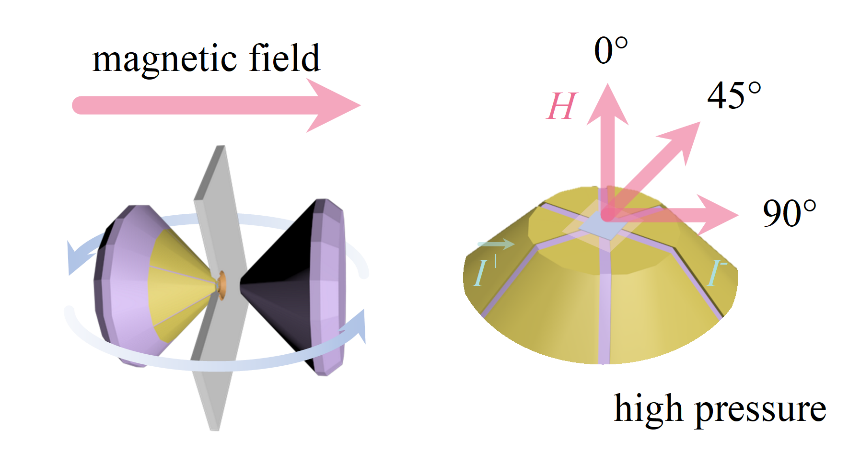


Figure S3 Schematic illustration of setup for high pressure angular-dependent hall resistance measurement. A rotational sample holder was inserted into the sample chamber and the electromagnet provide transverse magnetic field up to 1.1 T. By rotating the sample holder, we can change the direction between magnetic field and the normal direction of sample surface.





Figure S4 (a) Room temperature angular dependent *R*_AHE_-*B* curves and (b) the corresponding *θ*_M_-*θ*_H_ curve at 21 GPa. *θ*_M_-*θ*_H_ curve falls in the top-left zone representing θ_M_ > θ_H_ with *K*_1_ < 0 (in-plane magnetic easy axis). The fitted magnetic anisotropy constant *K*_1_ is calculated to be -0.789 MJ/m^3^ by using the formula of *F* = *K*_1_ sin^2^θ_M_ − μ_0_*HM*cos (*θ*_H_ − *θ*_M_), where μ_0_*HM* is assumed 1 MJ/m^3^.

1. **Hall resistance and magnetoresistance at different temperatures and pressures**


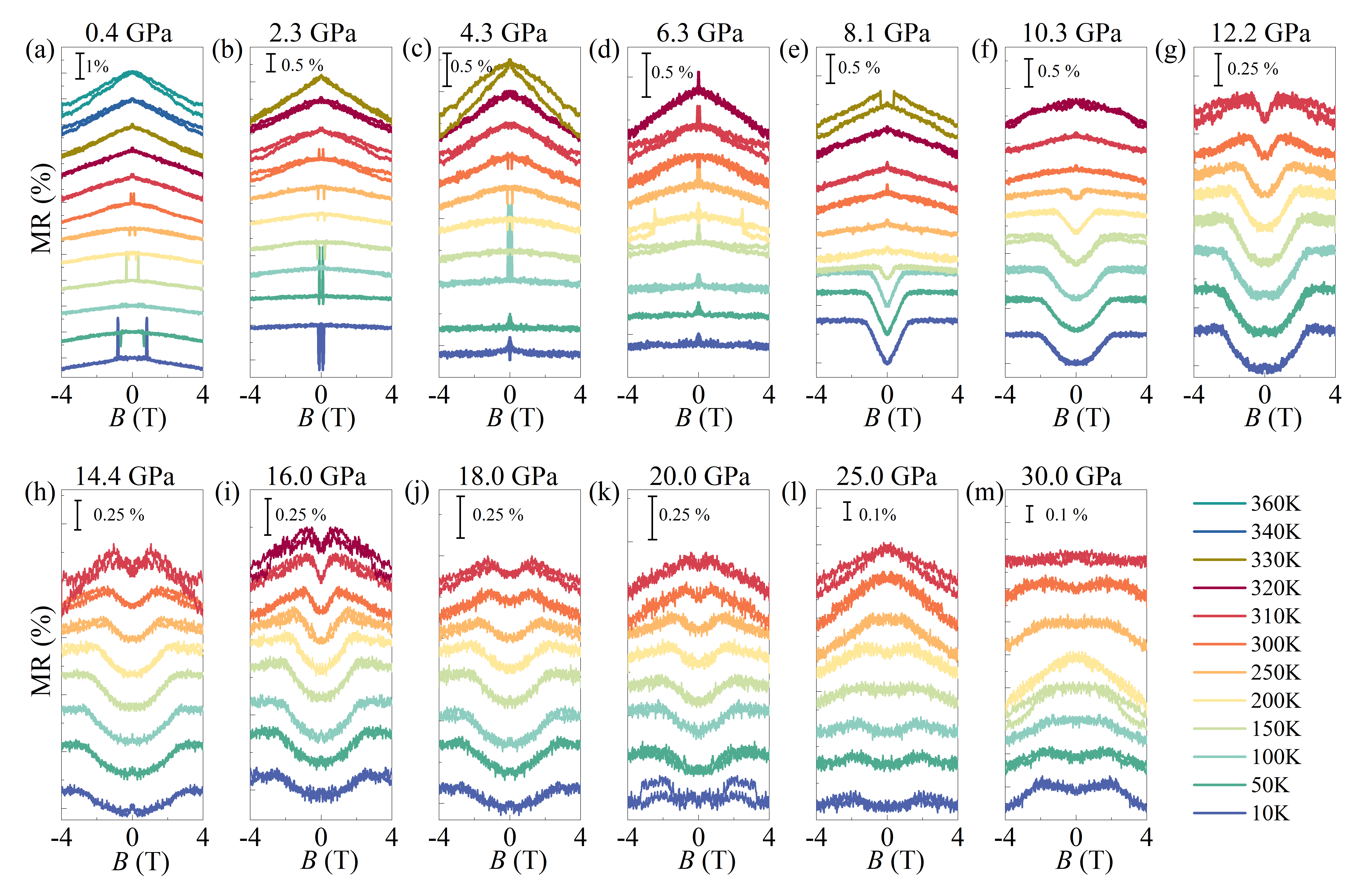


Figure S5 The isothermal magnetoresistance at various pressures.


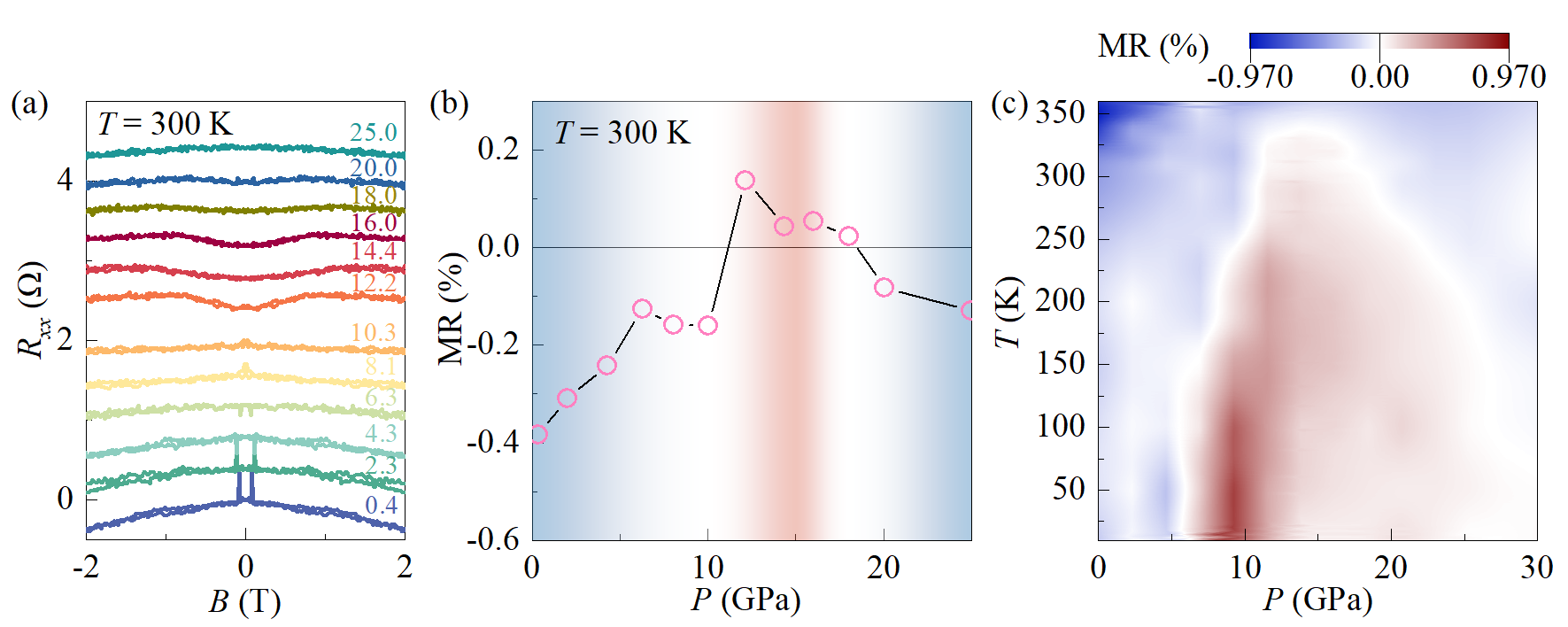


Figure S6 (a) Longitudinal resistance *R_xx_* as a function of magnetic field under various pressures at *T* = 300 K (b) Pressure dependent MR ratio ($MR=\frac{R_{2T}-R_{0}}{R_{0}}\times100\%$) at *T* = 300 K. The sign reversal occurred between 10.3 and 12.2 GPa, consistent with the *R*^s^/*R*^r^-*P*, *θ*_0 Oe_-*P* and *R*_H_-*P* curves, indicating the electronic structure evolution with pressure (c) Color plot of MR ratio at different pressures and temperatures.


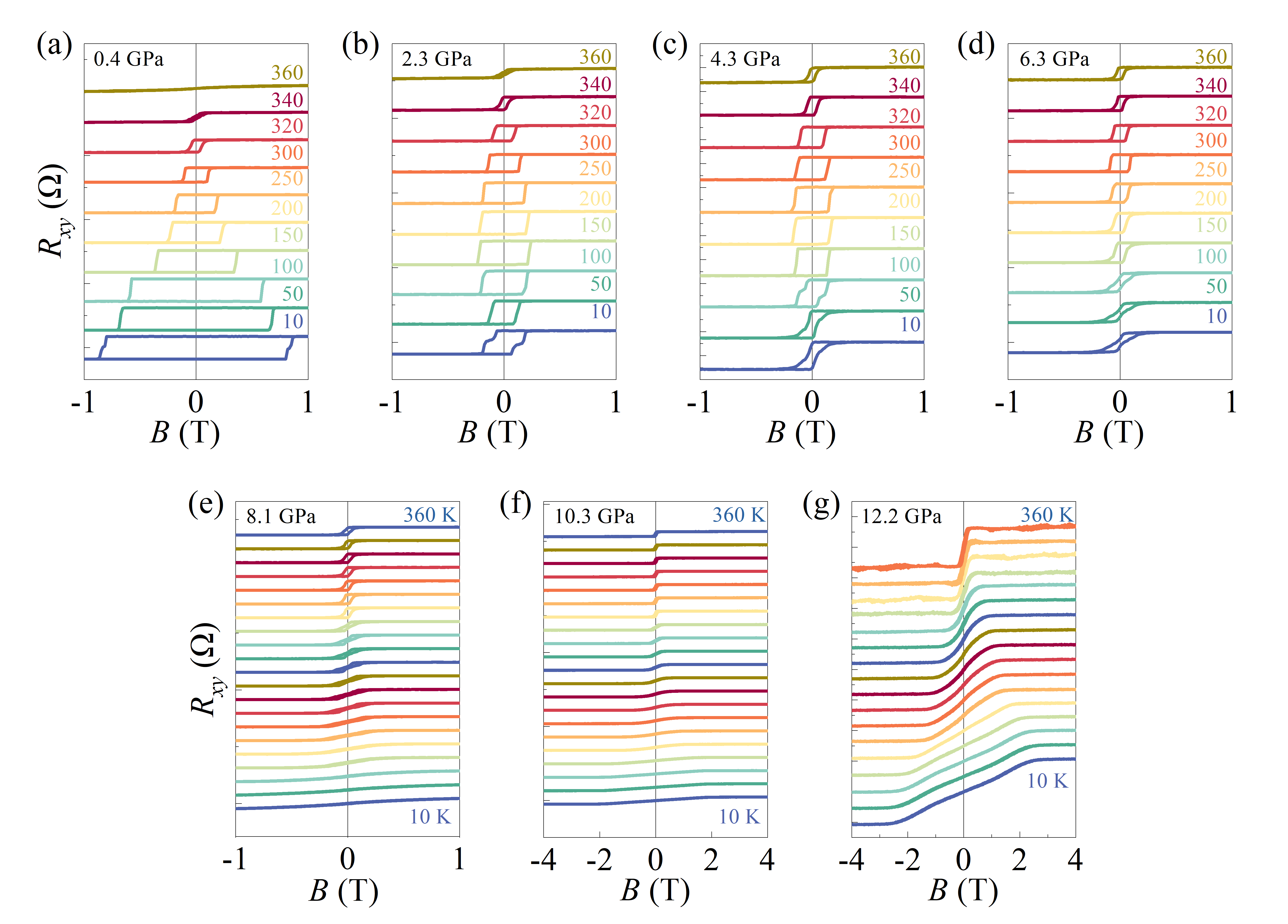


Figure S7 The magnetic field dependence of *R_xy_* at different pressures of (a) 0.4, (b) 2.3, (c) 4.3, (d) 6.3, (e) 8.1, (f) 10.3 and (g) 12.2 GPa.


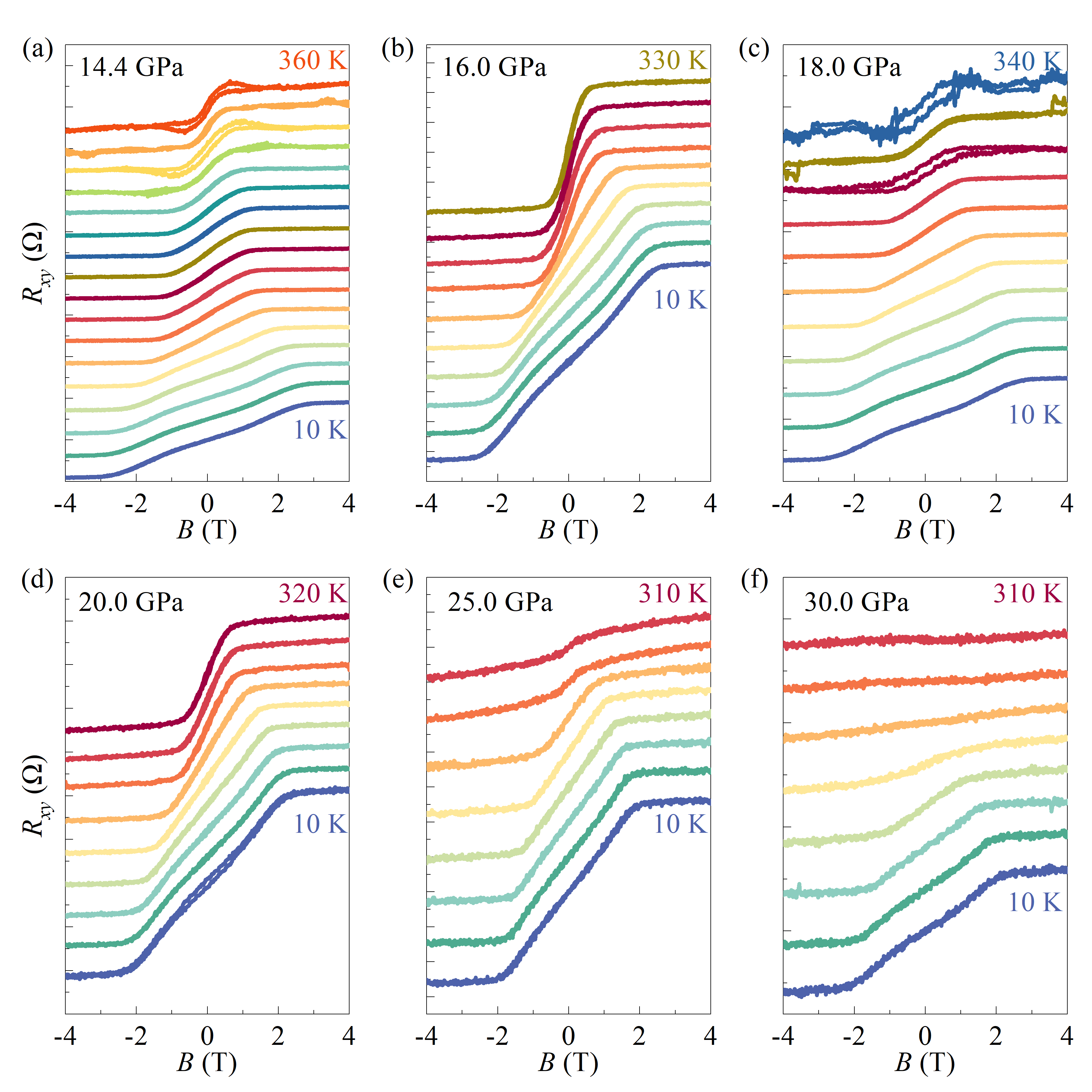


Figure S8 The magnetic field dependence of *R_xy_* at different pressures of (a) 14.4, (b) 16.0, (c) 18.0, (d)20.0, (e) 25.0, and (f) 30.0 GPa.

The pressure dependent magnetoresistance (MR) (defined as MR=(*R*(H)-*R* (0 T))/*R* (0 T)) of Fe_3_GaTe_2_ with magnetic field perpendicular to ab plane are presented in Figure S5. Detailed analyses of MR data are presented in Figure S6. Magnetoresistance can reflect features of magnetic phase transition and electronic structure. As shown in Figure S6a, at the initial pressure of 0.4 GPa, Fe_3_GaTe_2_ shows negative MR with the maximum value to be -0.75 % at *T* =300 K and B = ± 4 T. Moreover, the low field MR data, the negative MR features survive up to 10.3 GPa at *T* =300 K, above which the MR presents dip-like feature with the positive MR in the relatively low magnetic field region (negative MR in a wide magnetic field region). With further increasing the pressures, the magnetic regime of the dips in MR curves correspond well to the saturated magnetic field (*B*_s_) of *R_xy_*(*B*). The negative/positive MR seems to strongly associate with the spin-dependent charge carrier scattering (out-of-plane to in-plane transition). At the maximum pressure of 25.0 GPa, no dip features can be observed in the MR. To clearly see the MR evolution under high pressure, MR@2T were summarized in Figure S6b. Two sign reversal position can be marked as *P*_1_ (~12.2 GPa) and *P*_2_ (~ 20 GPa). Notably, *P*_1_ is also consistent with the critical pressure obtained from the anomalies in squareness ratio and *R*_H_ slope change, which indicate the reconstruction of Fermi surface contributing to the MR behaviors. The MR evolution at different pressures and temperature was plotted in Figure S6c. A dome like shape can be observed in Figure S6c, which is also depend on the MR field region we choose.

**6*.* Reproducibility of magnetic anisotropy evolution under pressure.**

To confirm the reproducibility of high-pressure magnetic phase evolution, magneto-transport measurement was performed on a different sample of Fe_3_GaTe_2_ (S3, Figure S9). The thickness of Fe_3_GaTe_2_ nanoflake is about 20 nm. Figure S10a shows the pressure dependent *R*_xy_-*B* curves at *T* = 300 K. At low pressures of 0.33 - 4.28 GPa, the coercive field gradually increases, indicating the enhancement of ferromagnetism and PMA. With further increasing the pressure, coercive field decreases. As shown in Figure S10b, at *P* = 6.24 GPa, below *T*_0_, *R*^r^/*R*^s^ derivate from 1. With further increasing temperature, *T*_0_ shift to higher temperature. The changes are consistent with data obtained from Run1. As to the pressure dependent *H*_c_-*T* curves, two anomalies on slop marked by *T*_1_ and *T*_2_ are presented in Figure S10c. *T*_1_ and *T*_2_ evolves with pressure and the trend are also consistent with Run 1. Notably, no hysteresis features can be observed for Run 3 at the pressure of 16.33 GPa, an indication of disappearance of ferromagnetism at *T* = 300 K (*T*_c_ < 300 K). The lower Curie temperature compared with that obtained from Run1 may due to the thinner sample thickness. In addition, the different in characteristic temperatures under pressures, such as *T*_0_, *T*_1_ and *T*_2_, may also attribute to the thickness effect.

To reveal the thickness-dependent phenomena of pressure effect on Fe_3_GaTe_2_, we have further analyzed the magnetotransport data of sample S3. Two important things related to the thickness effect need to be addressed as follows:

(1) Curie temperature of Fe_3_GaTe_2_ presents thickness-dependent characteristic. As shown in Figure, the sample S3 (20-40 nm) have a Curie temperature of 327 K and the thick sample S1 (50-100 nm) possesses a higher Curie temperature of 358 K, indicative of the decreasement of *T*_c_ with the decreasing of layer number. Our results are consistent with recent report on thickness-dependent ferromagnetism in Fe_3_GaTe_2_^[1]^. (2) Out-of-plane magnetic anisotropy was strengthened with the decrease of sample thickness. Square-shaped hysteresis loops in AHE behavior with a magnetic field applied along *c*-axis have been observed in atomically thin flakes of S1 and S3 as shown in FigureS11, indicting robust PMA in Fe_3_GaTe_2_ nanoflakes. The thinner sample S3 have a larger coercive field than the thick sample S1, indicating stronger PMA in thinner sample. Besides, as shown in Figure S10, the *R*^r^/ *R*^s^ =1 feature of S3 can persistent to pressures below 6.24 GPa, larger than the critical pressure of out-of-plane to in-plane transition in sample S1. It means that the thinner sample is more resistance to pressure and higher pressure is need to change out-of-plane magnetic anisotropy to in-plane magnetic anisotropy.


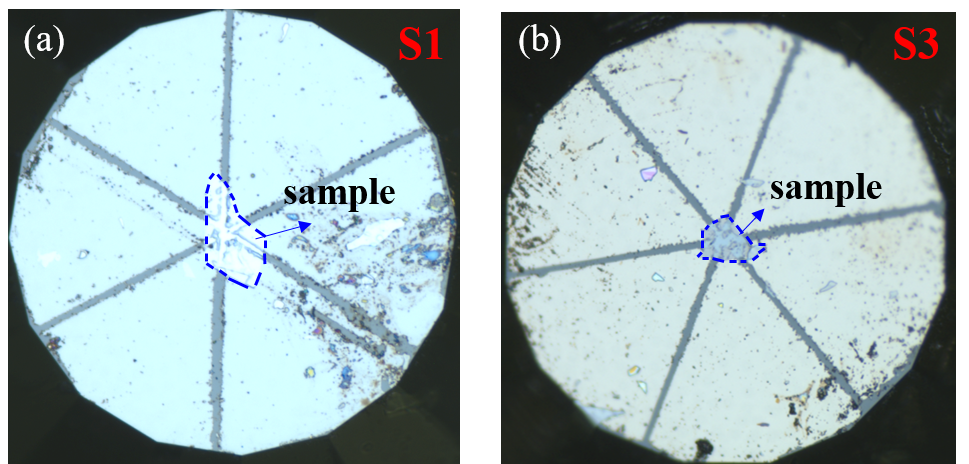


Figure S9 The optical image of (a) sample S1 and (b) sample S3 on Mo electrode. S3 is more transparent than S1, indicating a thinner thickness of S3.


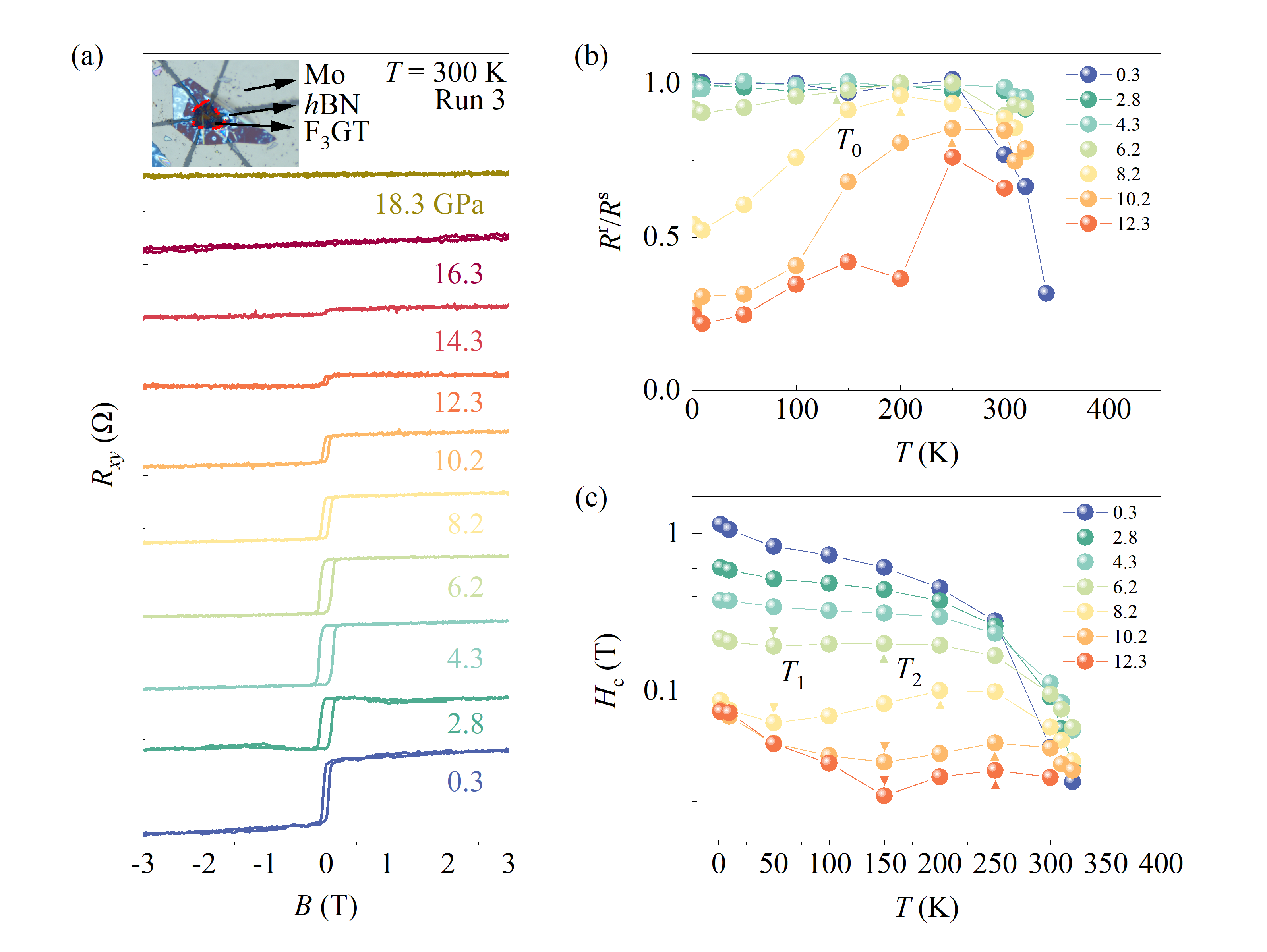


Figure S10 The high pressure magneto-transport data of different Fe_3_GaTe_2_ nanoflake for Run 3. (a) Magnetic field dependence of *R_xy_* under different pressures. (b) *R*^r^/*R*^s^ as a function of temperature under various pressure. (c) Temperature dependent coercive field at different pressures. The *H*_c_ (T) was presented in log scale to have a better view.





Figure S11 *R*_xy_-*B* curves for sample S1 (0.4 GPa) and S3 (0.33 GPa) at 10 K

**7*. T*_c_ determination**

By assuming the fitted *R*^2^*_xy_*-*B*/*R_xy_* curve should be parallel and spaced linearly in temperature, *T*_c_ can be estimated when the line become zero intercept via Arrot -plot approach. The critical isothermal magnetization around the Curie temperature can be described by Arrot-Nokes equation ^[2,3]^. By using hall resistance *R*_xy_ to replace *M*, *R*^2^*_xy_*−*B*/*R*_xy_ curves can be obtained.

Critical power law $\alpha{(1-T/T_{c})}^{\beta}$ fitting of *R*^s^_AHE_ -*T* was also applied to approximate *T*_c_ as presented in Figure S13. Typically, only the data in the vicinity of the PM to FM transition satisfy the power behavior. In our case, to have a reliable fitting *T*_c_ by using data derivate from critical transition, we assume the magnet satisfy the 3D XY model at all pressures and the critical exponent *β* was fixed to 0.35.

When fitting the curves, the *T*_c_ changes when choosing different critical exponent *β*, causing uncertainties in *T*_c_ estimation. Under this circumstance, we compare different *T*_c_s by fitting the curves with different *β*s for data obtained at 10.3 GPa as presented in Figure S14, where *β* = 0.3265 for 3D Ising model, *β* = 0.348 for 3D XY model, *β* = 0.369 for 3D Heisenberg model and *β* = 0.5 for mean field model ^[4]^. Generally, the fitted *T*_c_ value decreases with the decrease of critical exponent *β* value. The mean field theory seems to overestimate the *T*_c_ value, far above the *T*_c_ obtained from 3D model. However, for the 3D model, the *T*_c_s only varies in the range of Δ*T* = 22 K for different critical exponent β values, where the fitted *T*_c_s for 3D Ising model, 3D XY model and 3D Heisenberg model are 476 K, 487 K and 498 K, respectively.


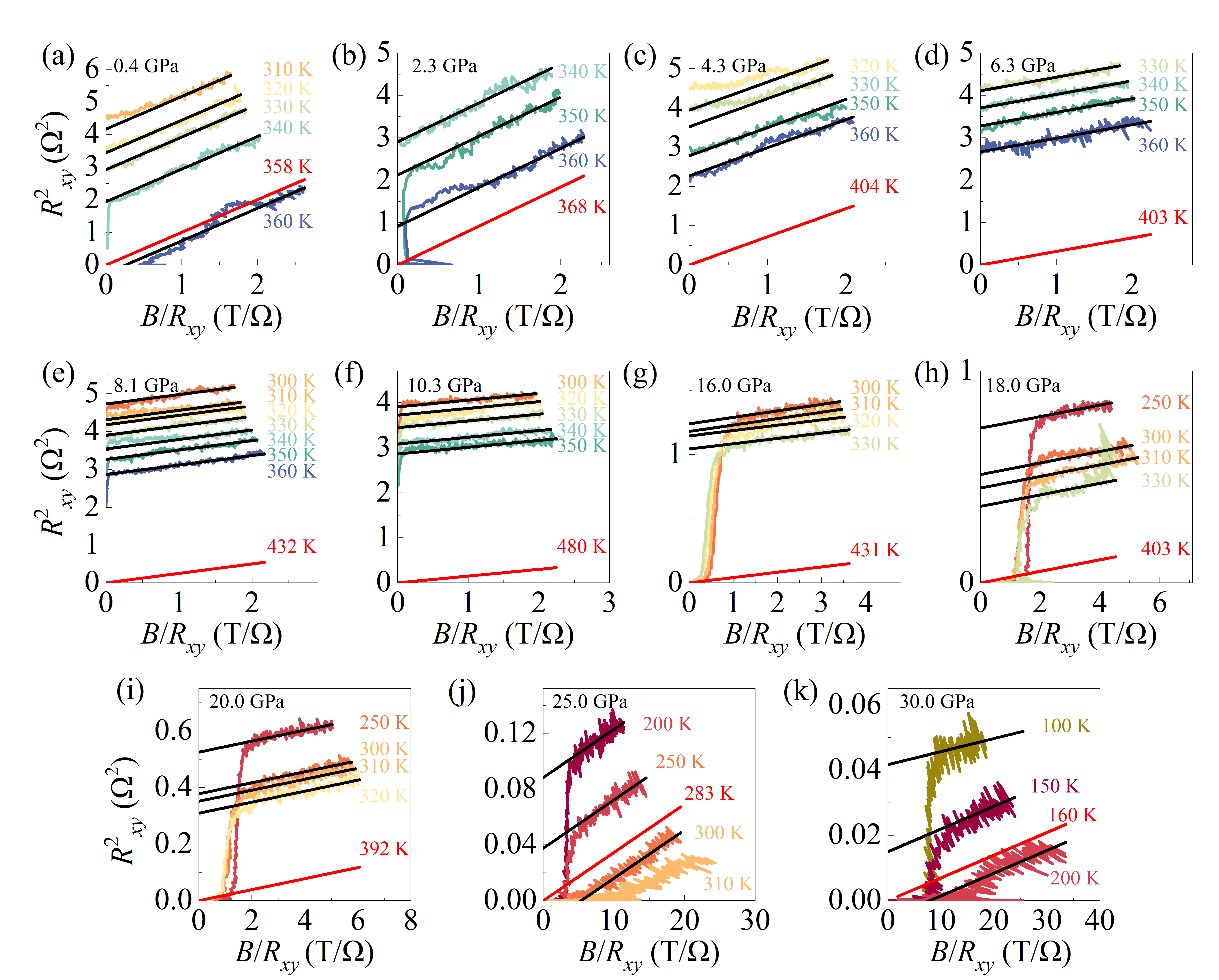


Figure S12 Arrot-plot method for determining curie temperature at different pressures.


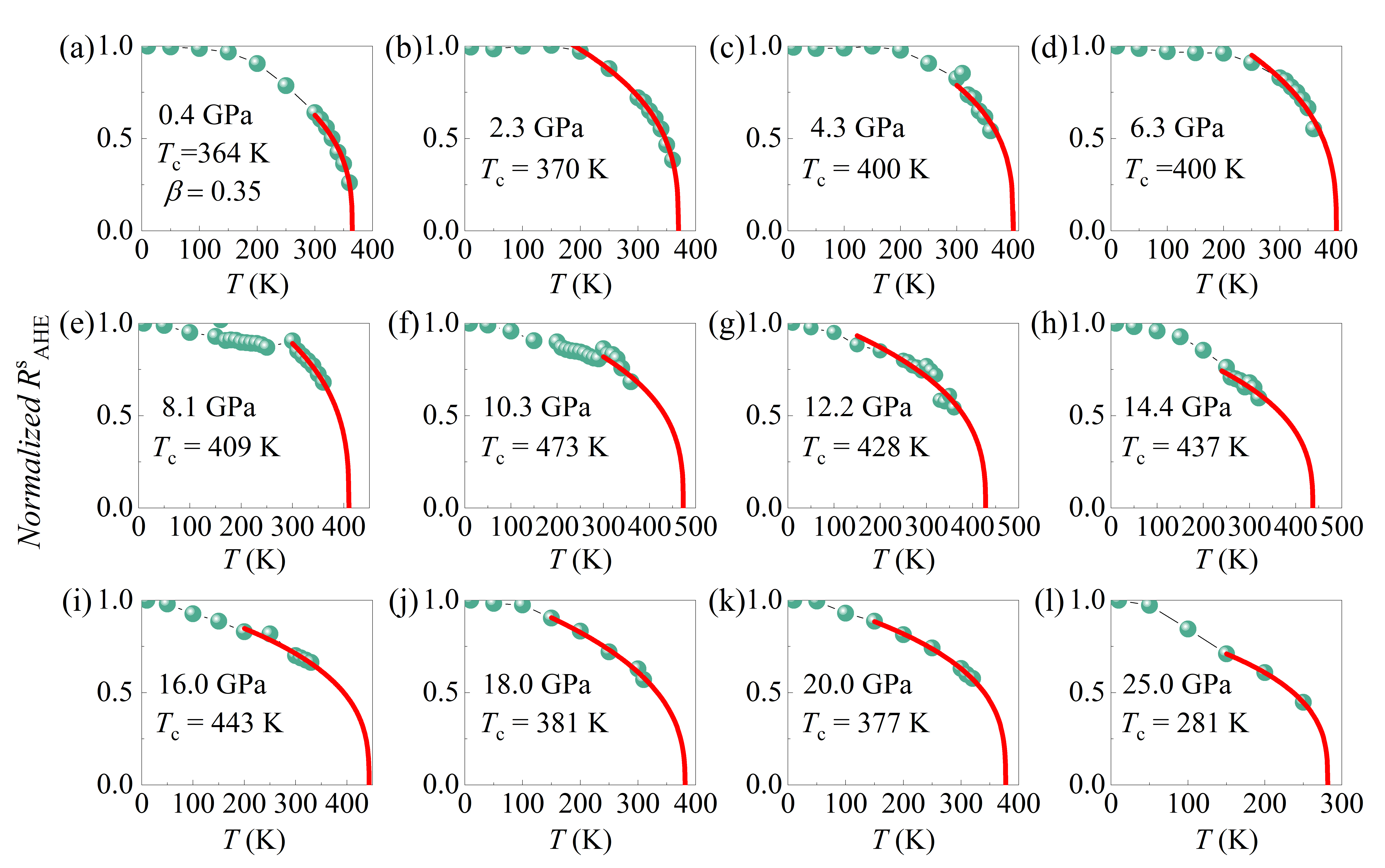


Figure S13 *R*^s^_AHE_-*T* curve fitting by the equation of $\alpha{(1-T_{c})}^{\beta}$at different pressures. To fit the curve, we assume the magnet satisfy the 3D XY model at all pressures and the critical exponent β was fixed to 0.35 when fitting the curve.


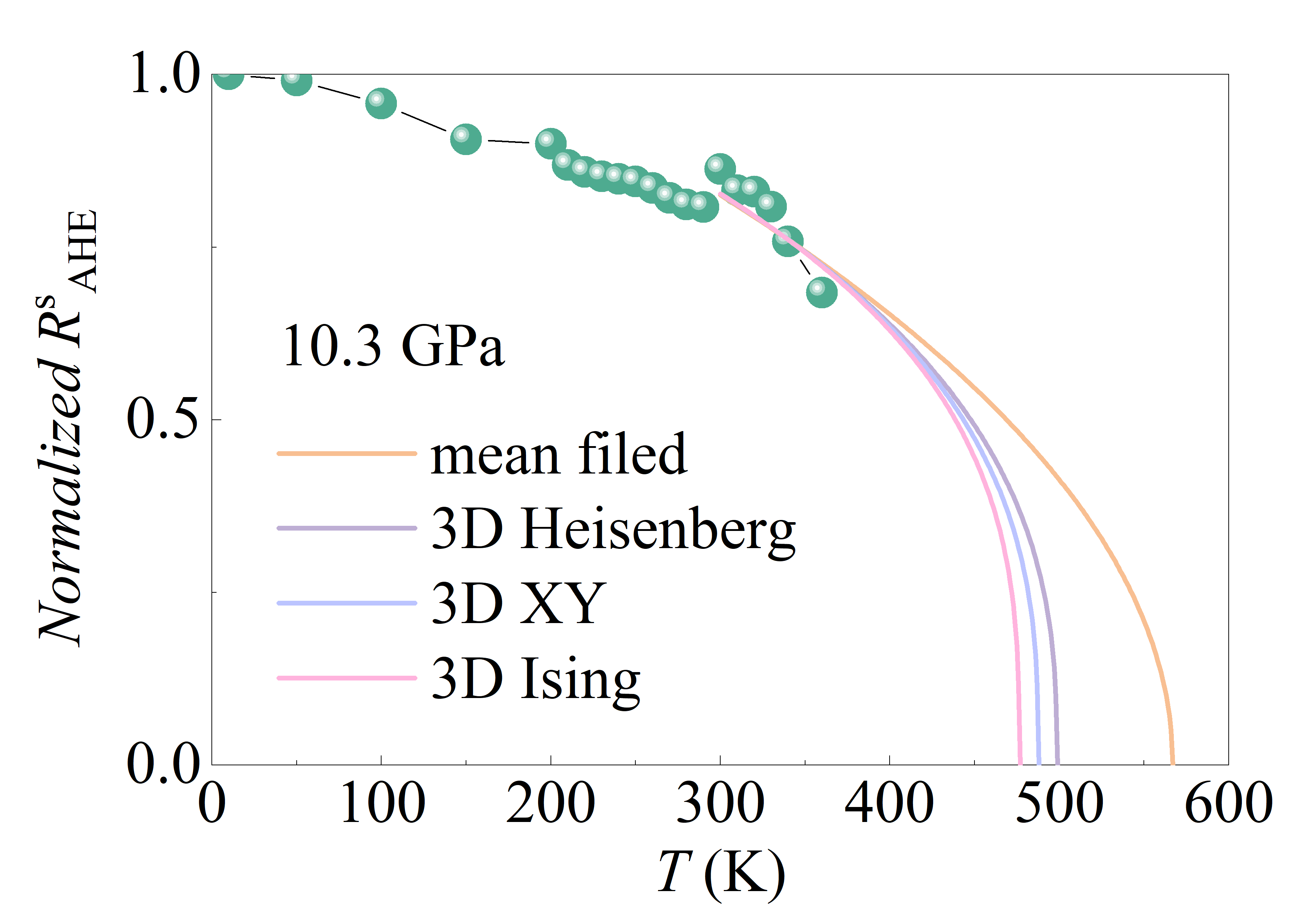


Figure S14 *R*^s^_AHE_-*T* curve fitting by the equation of $\alpha{(1-{(\frac{T}{T}}_{c}))}^{\beta}$at 10.3 GPa by using mean field model, 3D Heisenberg, 3D XY and 3D Ising model, respectively.

**8. High pressure Raman spectroscopy**


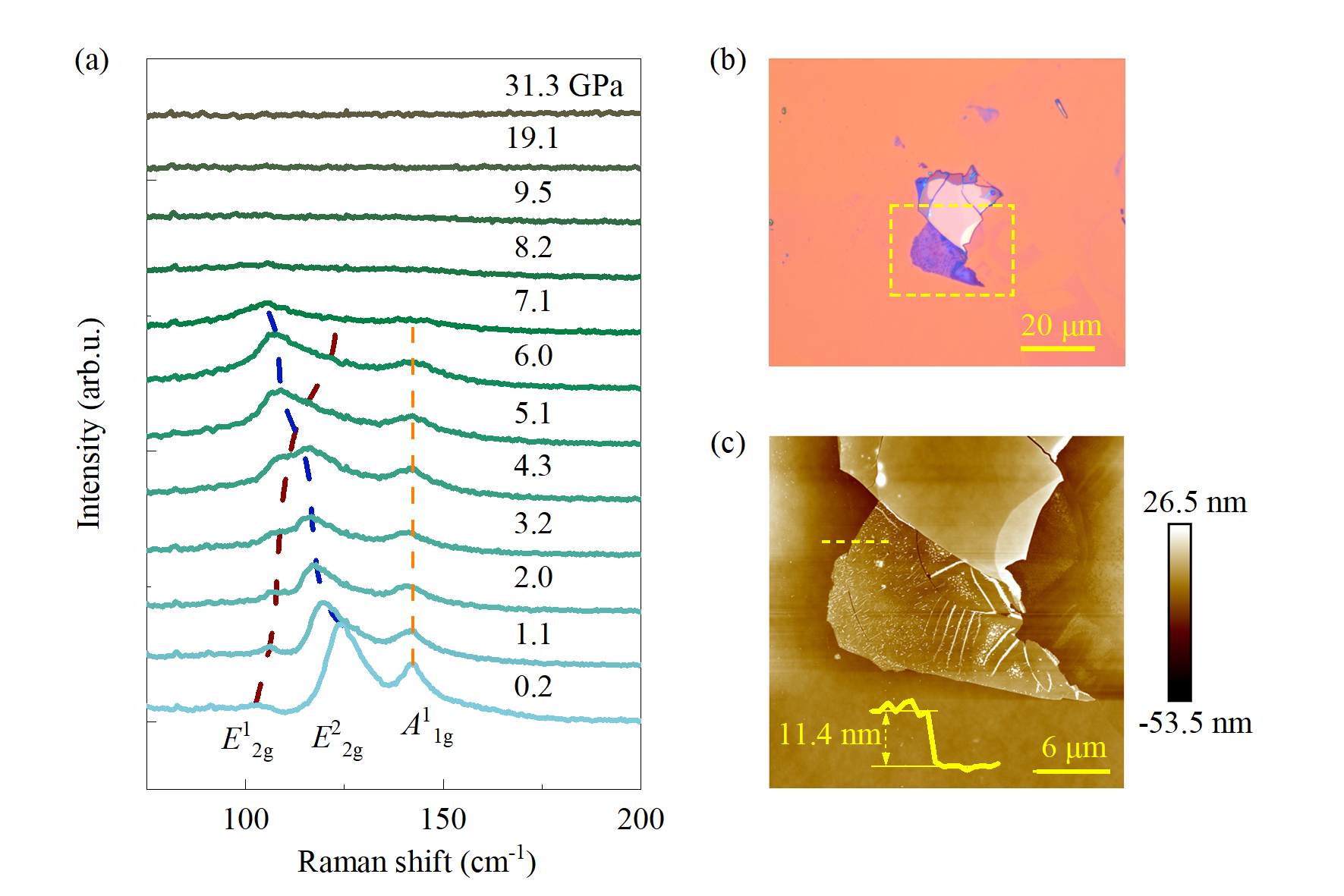


Figure S15 (a) Raman spectroscopy of Fe_3_GaTe_2_ under various pressures. (b) Optical image and (b) AFM image of Fe_3_GaTe_2_ nanoflake (thickness: 11.4 nm) for high pressure Raman measurement.

**9. Magnetic anisotropy energy calculation**


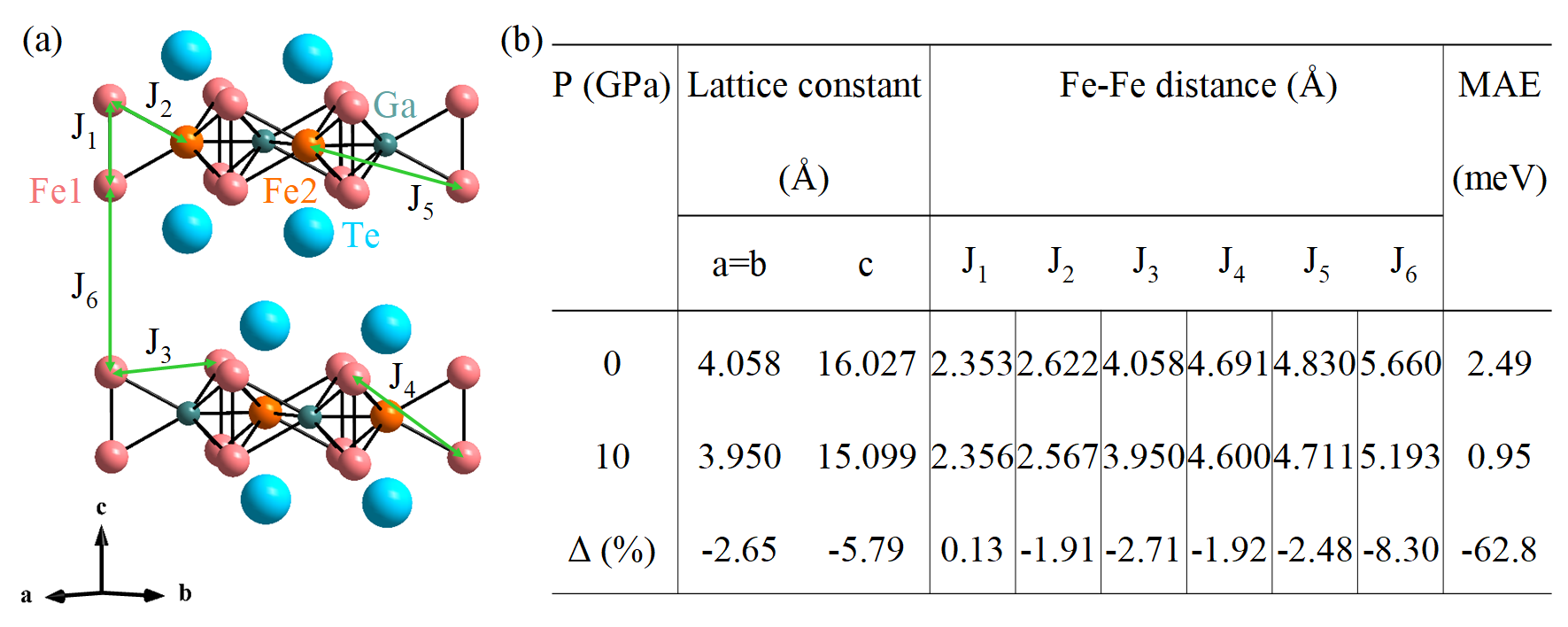


Figure S16 (a) Fe-Fe distance schematic corresponding to magnetic exchange interactions J_1_-J_6_. (b) The experiment lattice constants at 0 GPa and 10 GPa, the Fe-Fe distances, MAE defined as the energy difference between in-plane and out-of-plane magnetic configurations. Δ, the respective percentage changes.

**References**

[1] M. Wang, B. Lei, K. Zhu, Y. Deng, M. Tian, Z. Xiang, T. Wu and X. Chen, *npj 2D Mater. Appl.* **2024**, 8, 22.

[2] A. Arrott, J.E. Noakes, *Phys. Rev. Lett.* **1967**, 19, 786.

[3] A. Arrott, *Phys. Rev*. **1957**, 108, 1394.

[4] B. Liu, Y. Zou, S. Zhou, L. Zhang, Z. Wang, H. Li, Z. Qu and Y. Zhang, *Sci. Rep.* **2017**, **7**, 6184.
